# Prevalence and Correlates of High Stress and Low Resilience among Teachers in Three Canadian Provinces

**DOI:** 10.3390/jcm13154339

**Published:** 2024-07-25

**Authors:** Belinda Agyapong, Raquel da Luz Dias, Yifeng Wei, Vincent Israel Opoku Agyapong

**Affiliations:** 1 Department of Psychiatry, Faculty of Medicine and Dentistry, University of Alberta, Edmonton, AB T6G 2H5, Canada; 2 Department of Psychiatry, Faculty of Medicine, Dalhousie University, Halifax, NS B3H 2E2, Canada

**Keywords:** resilience, stress, Wellness4Teachers, teachers, prevalence, predictors

## Abstract

**Objective:** High stress levels can be problematic for teachers and indirectly affect students. Resilience may be a protective factor for overcoming stress. Knowledge about the prevalence and correlates of high stress and low resilience will provide information about the extent of the problem among teachers in Canada. **Methods:** This is a cross-sectional study among teachers in Alberta, Nova Scotia, and Newfoundland and Labrador in Canada. Participants self-subscribed to the Wellness4Teachers supportive text messaging program and completed the online survey on enrollment. Baseline data collection occurred from 1 September 2022 to 30 August 2023. Resilience and stress were, respectively, assessed using the Brief Resilience Scale (BRS) and the Perceived Stress Scale (PSS-10). The data were analyzed with SPSS version 28 using chi-squared tests and binary logistic regression analysis. **Results:** A total of 1912 teachers subscribed to the Wellness4Teachers program, and 810 completed the baseline survey, yielding a response rate of 42.40%. Most of the participants, 87.8%, were female, and 12.2% were aged 18 to 61 and above. The prevalence of low resilience was 40.1%, and high stress had a prevalence of 26.3%. After controlling for all the other variables in the logistic regression model, participants with low resilience were 3.10 times more likely to experience high-stress symptoms than those with normal to high resilience (OR = 3.10; 95% CI: 2.18–4.41). Conversely, participants who reported high stress were 3.13 times more likely to have low resilience than those with low to moderate stress (OR = 3.13; 95% CI: 2.20–4.44). Additionally, junior and senior high school teachers were, respectively, 2.30 times (OR = 2.30; 95% CI: 1.25–4.23) and 2.12 times (OR = 2,12; 95% CI: 1.08–4.18) more likely to have low resilience compared to elementary school teachers. **Conclusions:** Our study findings suggest a high prevalence of stress and low resilience among teachers in the three Canadian provinces. Administrators, policymakers in the educational field, school boards, and governments should integrate stress management and resilience-building strategies into teachers’ training and continuing professional development programs.

## 1. Introduction

Stress is an adaptive response to homeostatic imbalance; hence, stress response is a vital physiological response [1]. However, persistent exposure to stress may harm an individual’s health and general well-being [1]. The teaching profession is considered a social and interpersonally oriented occupation, with interactions and demands from parents, students, colleagues, and administrators and often a heightened work overload, with limited acknowledgment for accomplishments [2,3]. The role teachers play is thus complex and diverse, ranging from curriculum delivery to teaching children social skills and being carers and counsellors at school, which may all add to the stress they experience [4]. Stress can be defined as experiences of unpleasant, negative emotions like anger, frustration, nervousness, anxiety, and depression [5]. Generally, work stress negatively predicts teachers’ well-being [6]. A cross-sectional study among teachers reported a high-stress prevalence of 34.0%, and variables like teaching experience, age, and psychological job demands were significantly associated with stress [7]. Workload, student behaviour, and classroom management challenges have also been associated with teachers’ stress and may lead to burnout, low job satisfaction, and poor health in teachers [3]. Teacher stress has been associated with adverse consequences, including teacher attrition, absenteeism, burnout, and poor performance, which indirectly affect the students they teach [8,9]. A study among teachers in a public school showed that physiological and psychological factors threaten health, and the stress experienced by teachers in the workplace was associated with a high occurrence of physical ailments and somatic complaints [10]. Teachers’ perceived stress has also been positively correlated with their intention to leave the profession and negatively correlated with job satisfaction [11]. A strong correlation has also been reported to exist between stressful life issues and psychological illnesses such as anxiety and depression [12,13]. Additionally, stress-related disorders are prevalent, incur high economic losses, suffering, disability, and increased sick leave, and are associated with clinically significant psychological distress [14]. High levels of stress can also increase the risk of diabetes mellitus and atherosclerosis and have a significant effect on an individual’s immune system [12].

The COVID-19 pandemic imposed some challenges on teachers, particularly the readjustment in the mode of curriculum delivery and the increased use of technology. During this transition to remote instruction, more than 50% of teachers felt ineffective in the classroom and reported their inability to deliver the curricula as required to all classes [15]. In a systematic review, it was reported that the risk factors for increased incidence of psychological issues during the COVID-19 pandemic included difficulty in adapting to the distance education model and family/work conflict. The review also reported increased mental health issues such as generalized anxiety disorders and depression, as well as burnout syndrome, among teachers [16].

Resilience can be explained as the result of a dynamic relationship between individual risk and protective factors for stress [17]. It is the ability to overcome stress or adversity and achieve positive results [18]. Resilience also involves the interaction between biological, personal, and environmental or systemic sources and encompasses good adaptation or the capacity to sustain or regain psychological health irrespective of encountering adversity [19]. Individuals who are resilient have the ability to rapidly regain their composure after a stressful experience [20]. The ability to bounce back or recover from stress is essential in the teaching profession. Teachers with high resilience may have the fortitude to withstand pressure and may not be adversely affected by stress [21]. Resilience is essential in the quest to reduce teachers’ stress and improve their well-being. Nonetheless, a study in Canada among school board employees reported that about 35.0% of respondents had low resilience, suggesting that continued exposure to stress may result in dysfunctional adaptation to situations [22]. One study reported a statistically significant negative association between social support and work stress and a statistically significant positive relationship between resilience and social support [23]. This study also noted that resilience mediated the relationship between the perception of social support and work stress. Considering the relation between stress and resilience, it is vital to ascertain and understand teachers’ stress and resilience levels in the Canadian context. The primary aim of this study is to examine the prevalence and correlates of perceived high stress and low resilience among teachers in the three participating provinces: Alberta, Nova Scotia, and Newfoundland and Labrador. Our specific research objectives included the following:

1. To determine the prevalence of high stress and low resilience among Alberta, Nova Scotia, and Newfoundland and Labrador teachers.
2. To determine the sociodemographic, work-related, and other potential correlates of perceived high stress and low resilience among teachers in Alberta, Nova Scotia, and Newfoundland and Labrador.

It is hypothesized that the prevalence of high stress and low resilience among teachers in three participating provinces will be comparable to that reported in other jurisdictions [3,24]. Similarly, it is also hypothesized that sociodemographic characteristics, including gender, marital status, and age, professional-related factors, such as years of teaching, and other factors, such as level of perceived stress, will have independent predictive associations with low resilience and resilience will have independent predictive associations with perceived high stress among teachers.

## 2. Methodology

### 2.1. Study Design

This study adopted a quantitative cross-sectional survey design. Quantitative data were collected using web-based administered questionnaires through the University of Alberta’s REDCap platform [25], a secure web application for managing and building web-based surveys and databases.

### 2.2. Study Settings

This study was undertaken in Nova Scotia, Alberta, and Newfoundland and Labrador. Alberta has an approximate population of 4,756,408 and about 32,523 educators [26,27]. Nova Scotia and Newfoundland and Labrador are Eastern provinces of Canada, with population estimates of 1,066,416 and 540,418, respectively, according to Statistics Canada [27]. More than 10,000 public school teachers, support staff, and Community College faculty are represented by the Nova Scotia Teachers Union (NSTU) [28]. In Newfoundland and Labrador, the English School District oversees more than 250 schools, including 5 alternate sites, about 63,000 students, and more than 10,000 workers [29].

### 2.3. Data Collection and Outcome Measures

#### 2.3.1. Data Collection

Baseline data were collected from subscribers of the Wellness4Teachers program, which was launched at the beginning of the 2022/2023 academic year. The Wellness4Teachers application delivered a one-way (non-interactive) psychological intervention text message based on the concepts of cognitive behavioral therapy to the mobile phones of teachers who signed up for the program. Alberta, Nova Scotia, and Newfoundland and Labrador teachers were invited to self-subscribe to the Wellness4Teachers program [24]. This was undertaken through an advertisement organized in collaboration with the Alberta Teachers Association, the Alberta Catholic School Boards, the (NSTU), and the Newfoundland and Labrador Teachers Union (NLTU). Teachers could subscribe to the Wellness4Teachers program by texting “TeachWell” to a designated phone number to be automatically subscribed to receive supportive text messages.

All teachers were eligible and could subscribe to the program voluntarily. Subscribing teachers were invited to complete the web-based baseline surveys on enrollment via a survey link delivered via text message. Web-based baseline questionnaires were designed to collect demographic (age, sex, ethnicity, and marital status), professional (teaching experience, class size, major role, area of specialization, major source of stress), and clinical (stress and resilience) variables. The time needed to complete the survey questionnaire was about 5–10 min, and completion of the baseline survey was not dependent on receiving the daily supportive text messages. Teachers were also able to opt out of the program at any time by texting “stop” to the designated phone number. As the Wellness4Teachers program participation was based on self-subscription, educators and non-educators alike across Canada could subscribe to the program, although only data on subscribers who identified as educators in the three participating provinces are reported in this study.

#### 2.3.2. Outcome Measures

Clinical outcomes were evaluated using validated screening scales for self-reported symptoms. Although self-reported scales are not diagnostic tools, they can identify risk factors and early symptoms of potential mental distress. Resilience was measured using the six-item Brief Resilience Scale (BRS; mean scores of 1.00 to 2.99 suggest low resilience, 3.00 to 4.30 suggest normal resilience, and 4.31 to 5.00 suggest high resilience) [21]. Stress was assessed using the Perceived Stress Scale-10, a ten-item scale (PSS-10; scores ≥ 27 indicates likely high stress) [30,31,32]. Equally, a PSS-10 score of ≤13 indicates low stress, and moderate stress was denoted by scores of 14 to 26. The BRS and the PSS-10 have Cronbach’s α values of 0.78 and 0.85, respectively, signifying good internal consistency [32,33,34]. For prevalence estimates, the scales were studied as categorical variables. The primary outcome measures were the prevalence of high stress and low resilience at baseline in subscribers of Wellness4Teachers. The secondary outcome measures included correlates of high stress and low resilience.

#### 2.3.3. Sample Size Estimation

With approximately 55,000 teachers in Alberta, Nova Scotia, and Newfoundland, a sample size of 594 was needed for prevalence estimates for high stress and low resilience among teachers in the three provinces with a 95% confidence interval and a 4% margin of error.

### 2.4. Statistical Analysis

The data were analyzed using SPSS (version 28, I.B.M. Corp, NY, USA) [35]. Descriptive statistics were summarized for the demographic, professional, and clinical variables, including prevalence estimates based on the rural or urban location of the respondent’s school. The chi-square test/Fisher’s exact test was used to explore the associations between each of the demographic, clinical, and professional variables. Descriptive characteristics were presented as numbers and percentages, and a 2-tailed *p* value ≤ 0.05 was used to assess the statistical significance for all analyses. Two independent binary logistic regression analyses were used to identify variables that were independently predictive of likely high stress and low resilience. The regression model included variables that had a significant (*p* < 0.05) or near-significant (0.1 ≥ *p* ≥ 0.05) association with likely high stress and low resilience in the chi-square test/Fisher’s exact test analysis. Correlational analysis was performed to exclude any strong intercorrelations (Spearman’s correlation coefficient of 0.7 to 1.0 or −0.7 to −1.0) among the predictors prior to running the regression analysis. During data cleaning, survey responses that did not have at least the PSS-10 completed and educators who did not indicate they lived in the participating provinces were deleted, and the analysis and results are based only on completed survey data.

### 2.5. Ethics Approval

Approval of the study was granted by the University of Alberta Ethics Review Board (Pro00117558) and the Dalhousie University Human Research Ethics Review Board (REB # 2023-6840). Informed consent was obtained from all study participants.

## 3. Results

Table 1 below summarizes the sociodemographic characteristics of the participants based on their school location (urban or rural). Table 1 shows that 1912 teachers subscribed to the Wellness4Teachers program and received the link to the baseline survey on 31 August 2023. Of these, 810 subscribers completed all the demographic and professional questions and at least the PSS-10, yielding an effective response rate of 42.40%. Of those included in the study, 491 (60.6%) identified their schools as being in urban locations, with most of the participants living in Alberta 570 (70.4%). Overall, 711 (87.8%) were females, 732 (90.4%) were Caucasian, 457 (56.4%) were aged between 41 and 60 years old, and 516 (63.7%) were married. Most of the respondents, 307 (37.9%), also indicated they had 20 years or less but more than 10 years teaching experience. Finally, most of the respondents, 453 (55.9%), indicated that workload was their primary source of stress.

**Figure 1:**
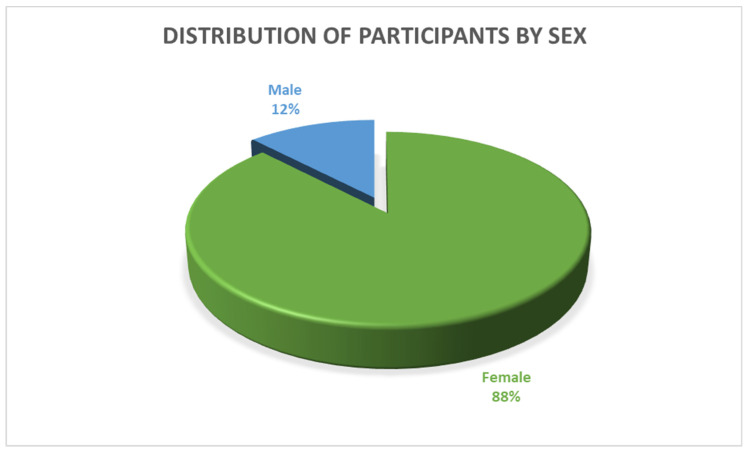
Distribution of participants by sex at birth.

Figure 1 shows the distribution of the participants by their sex at birth.

**Figure 2:**
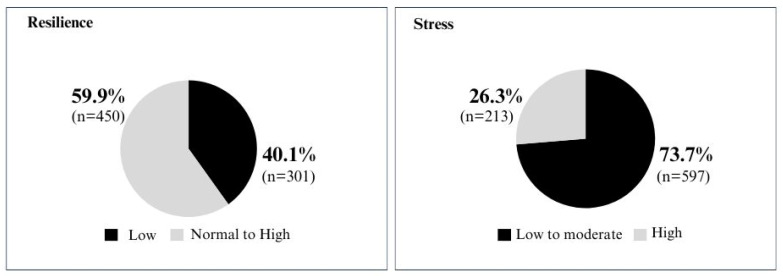
Overall prevalence of resilience and perceived stress levels.

Figure 2 below explores the overall prevalence of resilience and perceived stress levels. The data indicate that 59.9% of teachers exhibit normal to high levels of resilience, and 40.1% experience low resilience. In addition, 26.3% of educators presented with high stress.

The results were obtained through the Brief Resilience Scale (BRS) and the Perceived Stress Scale 10 (PSS-10), with N = 810 for the PSS-10 and N = 751 for the BRS.

The mean score and standard deviation (SD) for the PSS-10 were 22.33 and 6.09, respectively. For the BRS, the mean and SD were 3.13 and 0.90, respectively.

**Figure 3:**
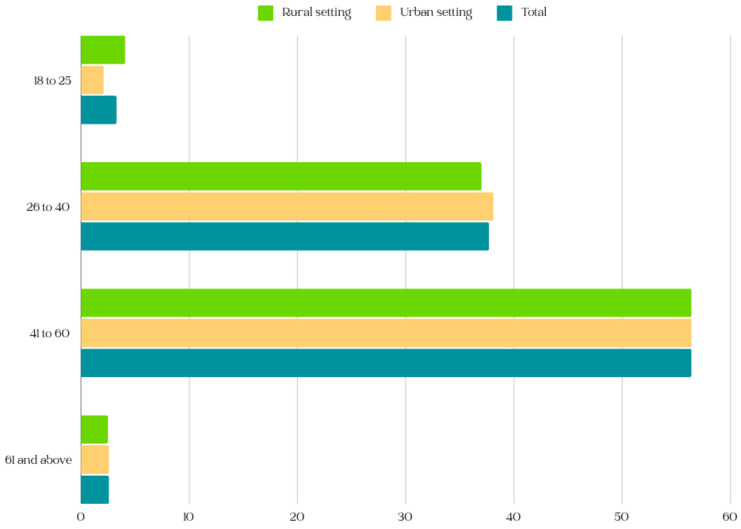
Distribution of participants by setting (rural or urban).

Figure 3 gives a visual distribution of the participants based on their settings (rural or urban).

### Univariate Analysis

Table 2 shows the outcomes of the chi-square test/Fisher’s exact test of the association between the demographic, professional, and clinical characteristics, as well as high stress and low resilience. Only five items were statistically significantly associated (*p* < 0.05) or had a near-significant association (0.1 ≥ *p* ≥ 0.05) with likely high stress. They include age, area of teaching specialization, number of years teaching, major role in the school, and low resilience. Participants aged 26 to 40 years old, junior high school teachers, English-specialized teachers, and teachers with between five and ten years of professional experience had high stress in comparison with other participants in the respective categories.

Similarly, six variables had a statistically significant (*p* < 0.05) or near-significant (0.1 ≥ *p* ≥ 0.05) association with low resilience, including age, number of children, number of years teaching, average class size, teachers’ major role, and high stress. Respondents aged 18 to 25 years, with one child, who were elementary school teachers who taught an average class size of 21–27 students, and who had between five and ten years of professional experience had low resilience compared to other participants in the respective categories.

Table 3 shows the logistic regression analysis to identify the correlates of high stress. Overall, five correlates that had a significant association (*p* < 0.05) or a trend toward a significant association (0.1 ≥ *p* ≥ 0.05) with high stress in the univariate analysis were added in the binary logistic regression model. The regression model was statistically significant, *Χ*^2^ (df = 18; n = 751) = 89.94 *p* = 0.000 < 0.0005, suggesting that the model could differentiate between participants with high stress and those with low to moderate stress. However, the model explained between 11.3% (Cox and Snell R^2^) and 16.4% (Nagelkerke R^2^) of the variance and correctly classified 73.2% of cases.

The results in Table 3 suggest that with a Wald value of 39.36, low resilience as a predictor of high stress made the most significant contribution to the regression model. In addition, only the “resilience” variable independently predicted the presence of high-stress symptoms in the participants. After controlling for all the other variables in the regression model, respondents who had low resilience were 3.10 times more likely to experience high-stress symptoms than those who did not (OR = 3.10; 95% CI: 2.18–4.41).

Although the variable of the teachers’ major role made no unique statistically significant contribution to the model, respondents who identified their major role as “other” were 0.355 times less likely to present with high stress than elementary school teachers when all the other factors were controlled. This implies that elementary school teachers were 2.82 (1/0.355) times more likely to present with symptoms of high stress compared to participants whose major role was “other” (OR = 2.82; 95% CI: 1.21–6.54).

Again, “area of teaching specialization” as a variable was not a significant contributor to the overall model. However, participants whose area of teaching specialization was English were 2.34 times more likely to present with symptoms of high stress compared to participants whose area of teaching specialization was Mathematics (OR = 2.34; 95% CI: 1.01–5.41) after controlling for all the remaining variables in the model. Again, participants whose area of teaching specialization was English were 3.58 times more likely to have high stress compared to participants whose area of teaching specialization was Science (Physics, Chemistry, or Biology) (OR = 3.58; 95% CI: 1.47–8.77) after controlling for all the remaining variables in the model. The other demographic and professional variables, like age and number of years teaching, did not independently predict the presence of high-stress symptoms in the participants.

Table 4 below shows the results of the logistic regression analysis to identify the correlates of low resilience among subscribers. Overall, six correlates that had a significant association (*p* < 0.05) or a near-significant association (0.1 ≥ *p* ≥ 0.05) with likely low resilience in the univariate analysis were included in the binary logistic regression model. The results for the logistic regression model for low resilience were statistically significant, *Χ*^2^ (df = 18; n = 751) = 101.41 *p* = 0.000 < 0.0005, suggesting that the model could differentiate between participants with low resilience and those with normal to high resilience. However, the model explained only between 12.6% (Cox and Snell R^2^) and 17.1% (Nagelkerke R^2^) of the variance and correctly classified 67.2% of cases.

The results in Table 4 show that with a Wald value of 40.34, “stress levels” made the most significant unique contribution to the regression model, followed by “teacher’s major role”, with a Wald value of 20.95. In addition, only two variables, “teachers’ major role” and “stress levels”, independently predicted the presence of low resilience in the participants. After controlling for all the other variables in the regression model, participants who reported being junior high school teachers as their major role were 2.30 times more likely to have low resilience than those whose major role was being an elementary school teacher (OR = 2.30; 95% CI: 1.25–4.23). Additionally, participants who reported “senior high school teacher” as their major role were 2.12 times more likely to have low resilience than those whose major role was “elementary school teacher” (OR = 2,12; 95% CI: 1.08–4.18). Similarly, participants who reported high stress were 3.13 times more likely to have low resilience than those whose did not (OR = 3.13; 95% CI: 2.20–4.44). Age as a variable did not significantly contribute to the model. However, participants aged 26–40 years old were 6.80 times more likely to have low resilience than those aged 18–25 years old (OR = 6.80; 95% CI: 1.13–40.97). Other demographics, such as the number of children, number of years teaching, and class size, did not independently predict the presence of likely low resilience in the participants.

## 4. Discussion

The primary aim of this study was to examine the prevalence and correlates of high stress and low resilience symptoms among subscribers of the Wellness4Teachers program in the three participating Canadian provinces: Alberta, Nova Scotia, and Newfoundland and Labrador. The study’s findings suggest that teachers in the three provinces may experience high levels of stress and low resilience.

The prevalence of high stress (26.3%) reported in this study is higher than the 7% prevalence of severe to extremely severe stress reported in a cross-sectional study among secondary school teachers in Klang, Malaysia [36]. Furthermore, two cross-national epidemiological studies among the general public in Australia and Canada reported an elevated distress prevalence of 11.1% and 12.0%, respectively [37], lower than the prevalence for high stress recorded in this study. In addition, a survey among employees in fifty-eight public and private sector organizations in the US, for instance, reported 9.6% moderate psychological distress [38]. This suggests that teachers in the three Canadian provinces may experience elevated stress compared to other employees and may be at a greater risk of other adverse health outcomes. Furthermore, teaching is categorized as one of the most high-stress occupations, which is evident in research reporting that 25% of teachers described the profession as very stressful or extremely stressful [39]. A higher prevalence (34%) of high stress was reported in another cross-sectional study among secondary school teachers prior to the COVID-19 pandemic [7]. This study found that age and the number of years of teaching were significantly associated with stress levels. On the contrary, they were not significantly associated with the stress levels in our current study, which may account for the disparity in the prevalence levels between the two studies. Additionally, a median prevalence for high stress of 32.5% was recorded in a scoping review of 70 studies on teacher stress [3]. This affirms that stress has always persisted among teachers. Although the COVID-19 pandemic may have impacted the prevalence of stress among the participants in the current study, there is no prior study specific to these provinces before the pandemic. A relatively high degree of stress (67%) was also recorded in a study [40] among primary/elementary school teachers during the twentieth century. A possible explanation for the comparatively lower prevalence of high stress in this study compared with the earlier study may be the improvement in professional development and advancement in technology, which may have enhanced the mode of curriculum delivery, making it more interactive and enjoyable for students and teachers. Technology has been used in the educational sector to enhance curriculum delivery, improve students’ learning by helping students understand certain concepts and skills, augment the curriculum, and transform class interactions with new pedagogy [41]. In addition, teachers are offered periodic professional development, and research shows that teachers who receive training, resources, and support to meet new challenges are less likely to experience high-stress symptoms [42]. Furthermore, though the COVID-19 pandemic era was generally difficult for teachers with the change to an online mode of curriculum delivery, a study report indicates that teacher trainees preferred online learning platforms over traditional learning strategies despite having less experience in knowledge and skills [43]. Further, there is research indicating that teachers’ high resilience positively impacted student learning outcomes during the pandemic when teaching online [44]. This highlights the importance of implementing evidence-based programs to help teachers become more resilient.

With a Wald value of 39.361, this study’s strongest predictor of high stress was the presence of low resilience symptoms among participants. Participants who had low resilience were 3.10 times more likely to experience high-stress symptoms compared to participants who had normal to high resilience. This finding adds to the literature and suggests that teachers with normal to high resilience can easily “bounce back” and may not be adversely affected by stressful situations compared to teachers with low resilience [21]. This finding corroborates other studies that reported that low resilience significantly predicted high stress among school teachers [42,45] and in the general population [46]. The results suggest that participants with high resilience can withstand low to moderate occupational stress levels. Thus, resilience is crucial in preventing or reducing high stress among educators. Resilience may invariably decrease the severity of mental health problems and improve educators’ well-being. A study during the pandemic also reported that resilience was an important factor and one of the strongest mediating effects, buffering the negative effects of perceived stress [47].

Although the area of teaching specialization as a variable did not make a statistically significant contribution to the regression model, participants whose area of teaching specialization was English were more than two times as likely to have high stress compared to participants whose area of teaching specialization was Mathematics. Again, participants whose area of teaching specialization was English were three and a half times more likely to have high stress than participants whose teaching specialization was Sciences (Physics, Chemistry, Biology). These findings support findings in a review article [48] which reported that in language learning settings, stress is considered to be the most common mental issue educators encounter in their work. On the contrary, other research has suggested that some students struggle in the fields of Science and Mathematics, which may be a source of stress for teachers, as they desire their students to excel [49,50].

Teachers’ major role as a variable did not make a statistically significant contribution to the model, and junior and high school teachers did not differ significantly in their stress levels in comparison with elementary school teachers. Research in the Netherlands suggests that more than half of the teachers working in primary education experience high levels of work stress, but this study did not report higher stress levels in junior or high school teachers [51]. In the current study, however, elementary school teachers were almost three times as likely to have higher stress than participants who indicated their major role as “other.” It is possible that those who indicated “other” as their major role have fewer responsibilities than elementary school teachers, which might explain the higher likelihood of the latter presenting with high stress.

The current study also indicates that age and teaching experience were not associated with stress, unlike in another study [7], where these factors and job demands were significantly associated with stress levels. Additionally, this study is contrary to our hypothesis and the other literature, which suggests that demographic and professional factors such as sex, age, marital status, and number of years teaching do have an association with high stress among teachers [3]. Again, this study did not find any statistically significant differences in the prevalence of high stress between rural and urban teachers, contrary to findings reported in another study [52] which indicated that urban school teachers experienced significantly more stress than their rural counterparts. It is noteworthy that previous studies examining correlates of high stress among educators may not have included resilience as a predictor, which may account for the differences in the findings between this study and previous studies.

The prevalence of low resilience was 40.1% in this study, which is slightly higher than the prevalence of low resilience among school board employees and the general public (35.0% and 37.4%, respectively) in the city of Fort McMurray, Alberta, Canada, which experienced wildfires in 2016 and flooding in 2020. [22,53,54].

This study shows that two variables, “high stress” and “teachers’ major role”, were the only independent correlates of low resilience. Participants who reported high-stress symptoms were three times more likely to have low resilience than those who had low- to moderate-stress symptoms. The present study is consistent with the outcome of a cross-sectional study where resilience scores were negatively correlated with the mean total score for stress [55]. Teaching as an interpersonal occupation implies that teachers influence the mental health trajectory of future generations through frequent interaction with students during their developmental phases [42]. Therefore, it is vital to reduce teachers’ stress levels and improve their mental well-being through resilience building. A systematic review reported that stress levels were high when resilience was low, reiterating that low stress and high resilience were better correlates of well-being [56].

A study conducted in Fort McMurray [54] identified age as a significant predictor of low resilience, with respondents who were older than 40 years old and those between 25 and 40 years old less likely to show low resilience compared to those younger than 25 years old. This is contrary to our current study findings, which suggest that although age as a variable did not make a significant contribution to the overall model, participants who were 26–40 years old were 6.80 times more likely to have low resilience compared to those aged 18–25 years old (OR = 6.80; 95% CI: 1.13–40.97). A possible explanation is that teachers aged 25–40 may have taught for some time and may have little support at this stage compared to the younger group, thus reducing their resilience. This possible explanation is supported by findings from another study which indicated that younger participants have higher resilience than older participants, and resilience positively correlates with social support [57]. This study did not find any significant difference in the prevalence of low resilience between rural and urban teachers, contrary to a study that reported that rural school teachers felt socially supported, perceived improvements in their work environment, and had a positive perception of professional recognition compared to urban school teachers [52,58] and thus may be expected to have a lower prevalence of low resilience.

In the current study, junior and senior high school teachers were both two times more likely to have low resilience compared with elementary school teachers. This finding is inconsistent with the results reported in this study for high stress. However, this finding contradicts previous research which suggests that elementary school teachers who teach younger children would be expected to have higher levels of stress and, by association, a higher prevalence of low resilience [59].

On the other hand, the adolescent years are transitional stages associated with physical, social, and psychological changes, which can translate into behavioral problems and poor academic performance [60]. Poor student performance or failure is sometimes construed as the responsibility of schools and teachers [61], which may increase stress levels and reduce resilience in junior and senior high school teachers.

Finally, other demographic and professional factors such as the number of children, number of years teaching, and class size did not independently predict the presence of likely low resilience in the participants, contrary to our hypothesis [24] and a previous study [62] which suggested that years of teaching positively correlates with higher resilience. It is also noteworthy that previous studies examining the correlates of low resilience among educators may not have included high stress as a predictor, which may account for the differences in the findings from this study and previous studies.

This study had some limitations. First, the scales used to assess stress and resilience, although standardized, are not diagnostic tools. However, the PSS-10 and the BRS have been widely used in studies assessing stress and resilience levels. Second, the response rate in this study, 42.4%, was low, though this was much higher than the response rates typically achieved in web-based surveys [63,64]. Even with the low response rate, the study sample size of 810 was much higher than the projected sample size of 594 for the prevalence estimates in the three Canadian provinces. Thus, the margin of error for the prevalence estimates for high stress was only 3%, which is lower than the projected 4% margin of error. Third, the demographic variables in the study may not mirror the demographics of teachers in Canada, as only teachers in three provinces were involved in the study. Furthermore, the study participants were mainly female, with only 12.2% male participants; although the teaching profession in Canada is disproportionately made up of females [65], males may have been slightly under-represented in this study. It is not possible to ascertain whether the male non-response rate was different from the response rate for female subscribers or whether males did not enroll in the Wellness4Teachers program at the same rate as female teachers. In addition, it is impossible to determine whether the demographic or clinical profiles of responders and non-responders to the survey were similar or dissimilar. Given the unknowns related to the non-responders, it is possible that potential differences between the respondents and non-respondents may have biased our study.

Thus, the study findings may not be generalizable across Canadian teachers. Finally, given the cross-sectional nature of this study, it is not possible to confirm a causal relationship between low resilience and high stress, which were included as correlates in the regression models predicting the other variables. At best, this study confirms an association between these two variables. In addition, it is possible that the inclusion of stress and resilience as correlates reduced the strength of some of the sociodemographic and work-related predictive variables. Despite these limitations, this study is the first to explore the prevalence and correlates of high stress and low resilience in the Canadian provinces and provides valuable insights into the health and well-being of Canadian teachers.

## 5. Conclusions and Implications for Policy and Practice

This study has highlighted the importance of resilience and its association with stress and teachers’ psychological well-being, and it reinforces how vital it is to implement resilience-building and stress reduction measures in teachers’ training and ongoing professional development programs.

Addressing the problem of high stress and building resilience among teachers can have a global positive effect on the school environment and students’ performance and well-being. Future research should explore additional variables that may predict high stress and low resilience. The study findings provide empirical data on stress and resilience among teachers, which may provide the basis for or serve as a reference point for future comparative and longitudinal studies in other educational contexts. Possible implications of the study findings are for the development of specific interventions that improve resilience and reduce stress among teachers. Mindfulness has been reported to be effective in promoting teachers’ well-being and positively impacting their personal and professional experience and could be adopted by school boards to support teachers [66]. Several other interventions have been proposed to improve teachers’ stress and general well-being, using mindfulness-based interventions combined with yoga or cognitive behavioral therapy (CBT) [67]. However, even under normal conditions, teachers are constantly under time constraints due to the stress associated with the demands of their jobs, making mindfulness and other face-to-face interventions challenging for some teachers and for whole schools to adopt. In line with this, it is suggested that education policy managers and governments incorporate evidence-based strategies that are cost-effective, are easily scalable, and do not require time or effort on the part of teachers to participate in or fit into their daily routines. One such program is the Wellness4Teachers program [24] in Canada, which was designed and adopted based on evidence gathered from randomized controlled trials [68,69], as well as evaluation of population-level ResilienceNHope interventions to mitigate stress, anxiety, depression, and post-traumatic stress disorder [70,71]. ResilienceNHope is an e-mental health application that sends one-way (non-interactive) cognitive behavioral-based therapy daily supportive messages by mobile text or email to support the psychological well-being of individuals and communities globally [72]. If the Wellness4Teachers text messaging program achieves positive outcomes, it may be a vital means of building resilience and reducing stress in teacher education programs. Administrators, policymakers in the educational field, school boards, and governments should also envision integrating in-service training on techniques for better classroom management and professional development focused on resilience building to help reduce the incidence of high stress and low resilience among impacted teachers.

## Figures and Tables

**Table 1:** Frequency distribution of sociodemographic, professional, and work-related variables based on school location (urban or rural).

Variables	Rural Setting n (%) N = 319	Urban Setting n (%) N = 493	Total n (%) N = 810
**Sociodemographic characteristics**			
**Age (years)**			
18–25	13 (4.1%)	14 (2.9%)	27 (3.3%)
26–40	118 (37.0%)	187 (38.1%)	305 (37.7%)
41–60	180 (56.4%)	277 (56.4%)	457 (56.4%)
61 and above	8 (2.5%)	13 (2.6%)	21 (2.6%)
**Sex at birth**			
Male	39 (12.2%)	60 (12.2%)	99 (12.2%)
Female	280 (87.8%)	431 (87.8%)	711 (87.8%)
**Provinces**			
Alberta	180 (56.4%)	390 (79.4%)	570 (70.4%)
Newfoundland	56 (17.6%)	47 (9.6%)	103 (12.7%)
Nova Scotia	83 (26.0%)	54 (11.0%)	137(16.9%)
**Relationship status**			
Single	42 (13.2%)	80 (16.3%)	122 (15.1%)
Married	210 (65.8%)	306 (62.3%)	516 (63.7%)
Common-law or partnered	49 (15.4%)	65 (13.2%)	114 (14.1%)
Separated or divorced	14 (4.4%)	33 (6.7%)	47 (5.8%)
Other	4 (1.3%)	7 (1.4%)	11 (1.4)
**Number of children**			
No child	92 (28.8%)	166 (33.8%)	258 (31.9%)
One child	43 (13.5%)	75 (15.3%)	118 (14.6%)
Two children	136 (42.6%)	174 (35.4%)	310 (38.3%)
Three children	32 (10.0%)	54 (11.0%)	86 (10.6%)
Four or more children	16 (5.0%)	22 (4.5%)	38 (4.7%)
**Ethnicity**			
Indigenous	11 (3.4%)	7 (1.4%)	18 (2.2%)
African descent	4 (1.3%)	5 (1.0%)	9 (1.1%)
East Asian	0 (0.0%)	12 (2.4%)	12 (1.5%)
Latino	0 (0.0%)	9 (1.8%)	9 (1.1%)
Middle Eastern	0 (0.0%)	5 (1.0%)	5 (0.6%)
South Asian	1 (0.3%)	8 (1.6%)	9 (1.1%)
Caucasian (European descent)	297 (93.1%)	435 (88.6%)	732 (90.4%)
Other ethnicities	6 (1.9%)	10 (2.0%)	16 (2.0%)
**Housing status**			
Own home	263 (82.4%)	408 (83.1%)	671 (82.8%)
Rented accommodation	45 (14.1%)	69 (14.1%)	114 (14.1%)
Living with family or a friend	11 (3.4%)	14 (2.9%)	25 (3.1%)
**Area of teaching specialization**			
English	57 (17.9%)	79 (16.1%)	136 (16.8%)
Mathematics	26 (8.2%)	35 (7.1%)	61 (7.5%)
Sciences (Physics, Chemistry, Biology)	17 (5.3%)	39 (7.9%)	56 (6.9%)
Arts (History, Geography, Social Studies, etc.)	27 (8.5%)	45 (9.2%)	72 (8.9%)
Music	10 (3.1%)	13 (2.6%)	23 (2.8%)
Physical Education	8 (2.5%)	13 (2.6%)	21 (2.6%)
Other	174 (54.5%)	267 (54.4%)	441 (54.4%)
**Teach only in your area of specialization**			
No	190 (59.6%)	274 (55.8%)	464 (57.3%)
Yes	129 (40.4%)	217 (44.2%)	346 (42.7%)
**Number of years teaching**			
5 years or less	45 (14.1%)	72 (14.7%)	117 (14.4%)
10 years or less but more than 5 years	44 (13.8%)	101 (20.6%)	145 (17.9%)
20 years or less but more than 10 years	124 (38.9%)	183 (37.3%)	307 (37.9%)
More than 20 years	106 (33.2%)	135 (27.5%)	241 (29.8%)
**Average classes size**			
20 or less	96 (30.1%)	72 (14.7%)	168 (20.7%)
21–27	177 (55.5%)	247 (50.3%)	424 (52.3%)
28 or more	46 (14.4%)	172 (35.0%)	218 (26.9%)
**School institution (type)**			
Catholic school	36 (11.3%)	116 (23.6%)	152 (18.8%)
Public school	277 (86.8%)	365 (74.3%)	642 (79.3%)
Other	6 (1.9%)	10 (2.0%)	16 (2.0%)
**Major role**			
Elementary school teacher	141 (44.2%)	218 (44.4%)	359 (44.3%)
Junior high school teacher	57 (17.9%)	92 (18.7%)	149 (18.4%)
Senior high school teacher	46 (14.4%)	74 (15.1%)	120 (14.8%)
Support staff	12 (3.8%)	22 (4.5%)	34 (4.2%)
Administrator	28 (8.8%)	42 (8.6%)	70 (8.6%)
Other	35 (11.0%)	43 (8.8%)	78 (9.6%)
**Source of stress**			
Workload	179 (56.1%)	274 (55.8%)	453 (55.9%)
Student behaviour	68 (21.3%)	101 (20.6%)	169 (20.9%)
Class size	14 (4.4%)	29 (5.9%)	43 (5.3%)
Lack of support from the school administration	25 (7.8%)	37 (7.5%)	62 (7.7%)
Other	33 (10.3%)	50 (10.2%)	83 (10.2%)

**Table 2:** Chi-square test of association between demographic, school-related, and role-related characteristics and likely stress and low resilience.

Variables	Low to Moderate Stress n (%) N = 597	High Stress n (%) N = 213	Chi^2^/Fisher Exact	*p* Value	Normal to High Resilience n (%) N = 450	Low Resilience n (%) N = 301	Chi^2^/Fisher Exact	*p* Value
**Sociodemographic characteristics**								
**Age (years)**			16.95	0.001			18.60	0.00
18–25	19 (70.4%)	8 (29.6%)			11 (44.0%)	14 (56.0%)		
26–40	203 (66.6%)	102 (33.4%)			152 (53.3%)	133 (46.7%)		
41–60	355 (77.7%)	102 (22.3%)			268 (63.8%)	152 (36.2%)		
61 and above	20 (95.2%)	1 (4.8%)			19 (90.5%)	2 (9.5%)		
**Sex at birth**			0.00	1.00			0.43	0.57
Male	73 (73.7%)	26 (26.3%)			58 (63.0%)	34 (37.0%)		
Female	524 (73.7%)	187 (26.3%)			392 (59.5%)	267 (40.5%)		
**Provinces**			3.15	0.21			2.62	0.27
Alberta	412 (72.3%)	158 (27.7%)			309 (58.6%)	218 (41.4%)		
Newfoundland	83 (80.6%)	20 (19.4%)			66 (67.3%)	32 (32.7%)		
Nova Scotia	102 (74.5%)	35 (25.5%)			75 (59.5%)	51 (40.5%)		
**Relationship status**			* 6.42	* 0.16			* 7.22	* 0.12
Single	96 (78.7%)	26 (21.3%)			57 (51.4%)	54 (48.6%)		
Married	385 (74.6%)	131 (25.4%)			295 (61.1%)	188 (38.9%)		
Common-law or partnered	75 (65.8%)	39 (34.2%)			60 (58.8%)	42 (41.2%)		
Separated or divorced	32 (68.1%)	15 (31.9%)			30 (65.2%)	16 (34.8%)		
Other	9 (81.8%)	2 (18.2%)			8 (88.9%)	1 (11.1%)		
**Number of children**			2.45	0.66			7.89	0.10
No child	188 (72.9%)	70 (27.1%)			132 (56.4%)	102 (43.6%)		
One child	83 (70.3%)	35 (29.7%)			59 (52.7%)	53 (47.3%)		
Two children	233 (75.2%)	77 (24.8%)			187 (64.7%)	102 (35.3%)		
Three children	67 (77.9%)	19 (22.1%)			53 (65.4%)	28 (34.6%)		
Four or more children	26 (68.4%)	12 (31.6%)			19 (54.3%)	16 (45.7%)		
**Ethnicity**			8.77	* 0.24			* 8.16	* 0.31
Indigenous	15 (83.3%)	3 (16.7%)			13 (76.5%)	4 (23.5%)		
African descent	9 (100.0%)	0 (0.0%)			4 (80.0%)	1 (20.0%)		
East Asian	7 (58.3%)	5 (41.7%)			4 (33.3%)	8 (66.7%)		
Latino	7 (77.8%)	2 (22.2%)			7 (77.8%)	2 (22.2%)		
Middle Eastern	3 (60.0%)	2 (40.0%)			3 (60.0%)	2 (40.0%)		
South Asian	7 (77.8%)	2 (22.2%)			4 (50.0%)	4 (50.0%)		
Caucasian (European descent)	540 (73.8%)	192 (26.2%)			408 (59.9%)	273 (40.1%)		
Other ethnicities	9 (56.3%)	7 (43.8%)			7 (50.0%)	7 (50.0%)		
**Housing status**			0.89	0.65			4.25	0.11
Own home	498 (74.2%)	173 (25.8%)			379 (60.6%)	246 (39.4%)		
Rented accommodation	80 (70.2%)	34 (29.8%)			56 (52.8%)	50 (47.2%)		
Living with family or a friend	19 (76.0%)	6 (24.0%)			15 (75.0%)	5 (25.0%)		
**School Situated**			0.26	0.63			1.29	0.29
Rural setting	232 (72.7%)	87 (27.3%)			183 (62.5%)	110 (37.5%)		
Urban setting	365 (74.3%)	126 (25.7%)			267 (58.3%)	191 (41.7%)		
**Area of teaching specialization**			12.03	0.06			4.80	0.57
English	89 (65.4%)	47 (34.6%)			72 (57.6%)	53 (42.4%)		
Mathematics	50 (82.0%)	11 (18.0%)			39 (73.6%)	14 (26.4%)		
Sciences (Physics, Chemistry, Biology)	48 (85.7%)	8 (14.3%)			30 (58.8%)	21 (41.2%)		
Arts (History, Geography, Social Studies, etc.)	52 (72.2%)	20 (27.8%)			40 (57.1%)	30 (42.9%)		
Music	16 (69.6%)	7 (30.4%)			13 (56.5%)	10 (43.5%)		
Physical Education	14 (66.7%)	7 (33.3%)			11 (57.9%)	8 (42.1%)		
Other	328 (74.4%)	113 (25.6%)			245 (59.8%)	165 (40.2%)		
**Teach only in your area of specialization**			0.93	0.38			0.01	0.94
No	336 (72.4%)	128 (27.6%)			260 (60.0%)	173 (40.0%)		
Yes	261 (75.4%)	85 (24.6%)			190 (59.7%)	128 (40.3%)		
**Number of years teaching**			14.40	0.002			9.21	0.03
Five years or less	85 (72.6%)	32 (27.4%)			58 (54.2%)	49 (45.8%)		
Ten years or less but more than five years	90 (62.1%)	55 (37.9%)			70 (51.5%)	66 (48.5%)		
20 years or less but more than 10 years	231 (75.2%)	76 (24.8%)			176 (61.3%)	111 (38.7%)		
More than 20 years	191 (79.3%)	50 (20.7%)			146 (66.1%)	75 (33.9%)		
**Average classes size**			1.95	0.38			4.67	0.10
20 or less	125 (74.4%)	43 (25.6%)			99 (63.9%)	56 (36.1%)		
21–27	319 (75.2%)	105 (24.8%)			221 (56.2%)	172 (43.8%)		
28 or more	153 (70.2%)	65 (29.8%)			130 (64.0%)	73 (36.0%)		
**School institution (type)**			1.72	0.40			3.79	0.15
Catholic school	118 (77.6%)	34 (22.4%)			74 (54.0%)	63 (46.0%)		
Public school	468 (72.9%)	174 (27.1%)			369 (61.6%)	230 (38.4%)		
Other	11 (68.8%)	5 (31.3%)			7 (46.7%)	8 (53.3%)		
**Major role**			18.40	0.002			38.92	0.00
Elementary school teacher	257 (71.6%)	102 (28.4%)			170 (51.1%)	163 (48.9%)		
Junior high school teacher	101 (67.8%)	48 (32.2%)			73 (54.5%)	61 (45.5%)		
Senior high school teacher	85 (70.8%)	35 (29.2%)			71 (64.0%)	40 (36.0%)		
Support staff	26 (76.5%)	8 (23.5%)			24 (75.0%)	8 (25.0%)		
Administrator	57 (81.4%)	13 (18.6%)			53 (81.5%)	12 (18.5%)		
Other	71 (91.0%)	7 (9.0%)			59 (77.6%)	17 (22.4%)		
**Source of stress**			2.14	0.71			5.84	0.21
Workload	336 (74.2%)	117 (25.8%)			256 (61.5%)	160 (38.5%)		
Student behaviour	129 (76.3%)	40 (23.7%)			83 (52.5%)	75 (47.5%)		
Class size	31 (72.1%)	12 (27.9%)			23 (57.5%)	17 (42.5%)		
Lack of support from the school administration	42 (67.7%)	20 (32.3%)			35 (60.3%)	23 (39.7%)		
Other	42 (71.1%)	24 (28.9%)			53 (67.1%)	26 (32.9%)		
**Resilience**			55.86	0.00	NA	NA	NA	NA
Normal to high resilience	374 (83.1%)	76 (16.9%)						
Low resilience	176 (58.5%)	125 (41.5%)						
**Stress**	NA	NA	NA	NA			55.82	0.000
Low to moderate stress					374 (68.0%)	176 (32.0%)		
High stress					76 (37.8%)	125 (62.2%)		

* Fisher’s exact value; NA—not applicable.

**Table 3:** Logistic regression model for stress.

	B	SE	Wald	df	Sig.	Exp(B)	95% CI for EXP(B)	
							Lower	Upper
**Age (years)**								
18–25			3.621	3	0.305			
26–40	0.283	0.544	0.271	1	0.603	1.327	0.457	3.855
41–60	−0.036	0.586	0.004	1	0.951	0.965	0.306	3.043
61 and above	−1.350	1.197	1.273	1	0.259	0.259	0.025	2.706
**Major role**								
Elementary school teacher			9.749	5	0.083			
Junior high school teacher	0.315	0.252	1.570	1	0.210	1.371	0.837	2.244
Senior high school teacher	0.325	0.285	1.302	1	0.254	1.384	0.792	2.418
Support staff	0.039	0.472	0.007	1	0.933	1.040	0.412	2.625
Administrator	−0.124	0.368	0.113	1	0.737	0.884	0.429	1.818
Other	−1.036	0.430	5.803	1	0.016	0.355	0.153	0.824
**Area of teaching specialization**								
English			10.140	6	0.119			
Mathematics	−0.850	0.427	3.967	1	0.046	0.428	0.185	0.987
Sciences (Physics, Chemistry, Biology)	−1.278	0.455	7.888	1	0.005	0.279	0.114	0.680
Arts (History, Geography, Social Studies, etc.)	−0.350	0.352	0.988	1	0.320	0.705	0.353	1.405
Music	−0.109	0.521	0.044	1	0.834	0.897	0.323	2.491
Physical education	−0.132	0.569	0.054	1	0.816	0.876	0.287	2.672
Other	−0.303	0.244	1.547	1	0.214	0.738	0.458	1.191
**Number of years teaching**								
5 years or less			3.049	3	0.384			
10 years or less but more than 5 years	0.406	0.327	1.547	1	0.214	1.501	0.791	2.849
20 years or less but more than 10 years	−0.009	0.327	0.001	1	0.979	0.991	0.522	1.882
More than 20 years	0.028	0.390	0.005	1	0.942	1.029	0.479	2.209
**Resilience**								
Low resilience	1.130	0.180	39.361	1	0.000	3.097	2.175	4.408
Constant	−1.398	0.532	6.890	1	0.009	0.247		

SE—standard error; B—beta; df—degrees of freedom; Sig—significance; CI—confidence interval.

**Table 4:** Logistic regression model for resilience.

	B	S.E	Wald	df	Sig.	Exp(B)	95% CI for EXP(B)	
							Lower	Upper
**Age (years)**								
18–25			4.467	3	0.215			
26–40	1.917	0.916	4.375	1	0.036	6.798	1.128	40.967
41–60	1.480	0.783	3.569	1	0.059	4.391	0.946	20.378
61 and above	1.339	0.762	3.084	1	0.079	3.815	0.856	16.999
**Number of children**								
No child			3.581	4	0.466			
One child	−0.339	0.414	0.669	1	0.414	0.713	0.316	1.605
Two children	−0.071	0.434	0.026	1	0.871	0.932	0.398	2.181
Three children	−0.474	0.401	1.395	1	0.238	0.623	0.284	1.367
Four or more children	−0.364	0.456	0.639	1	0.424	0.695	0.284	1.697
**Major role**								
Elementary school teacher			20.954	5	0.001			
Junior high school teacher	0.833	0.311	7.194	1	0.007	2.301	1.252	4.231
Senior high school teacher	0.753	0.345	4.750	1	0.029	2.123	1.079	4.179
Support staff	0.412	0.358	1.326	1	0.249	1.510	0.749	3.047
Administrator	−0.005	0.519	0.000	1	0.992	0.995	0.360	2.752
Other	−0.419	0.439	0.912	1	0.340	0.657	0.278	1.555
**Average class size**								
20 or less			2.861	2	0.239			
21–27	0.023	0.248	0.008	1	0.927	1.023	0.630	1.662
28 or more	0.294	0.204	2.068	1	0.150	1.342	0.899	2.003
**Number of years teaching**								
5 years or less			0.139	3	0.987			
10 years or less but more than 5 years	0.005	0.351	0.000	1	0.988	1.005	0.505	2.002
20 years or less but more than 10 years	0.088	0.302	0.084	1	0.772	1.092	0.603	1.974
More than 20 years	0.005	0.220	0.001	1	0.980	1.005	0.653	1.547
**Stress**								
High stress	1.142	0.180	40.338	1	0.000	0.319	0.225	0.454
Constant	−1.370	0.896	2.342	1	0.126	0.254		

## Data Availability

Data for this study are available upon reasonable request from the corresponding author.
